# Reconstruction and Exploratory Analysis of mTORC1 Signaling Pathway and Its Applications to Various Diseases Using Network-Based Approach

**DOI:** 10.4014/jmb.2108.08007

**Published:** 2022-01-08

**Authors:** Richa Buddham, Sweety Chauhan, Priyanka Narad, Puniti Mathur

**Affiliations:** Centre for Computational Biology and Bioinformatics, Amity Institute of Biotechnology, Amity University Uttar Pradesh Noida-201313, India

**Keywords:** mTORC1 network, differentially expressed genes, gene and pathway enrichment, survival analysis

## Abstract

Mammalian target of rapamycin (mTOR) is a serine-threonine kinase member of the cellular phosphatidylinositol 3-kinase (PI3K) pathway, which is involved in multiple biological functions by transcriptional and translational control. mTOR is a downstream mediator in the PI3K/Akt signaling pathway and plays a critical role in cell survival. In cancer, this pathway can be activated by membrane receptors, including the HER (or ErbB) family of growth factor receptors, the insulin-like growth factor receptor, and the estrogen receptor. In the present work, we congregated an electronic network of mTORC1 built on an assembly of data using natural language processing, consisting of 470 edges (activations/interactions and/or inhibitions) and 206 nodes representing genes/proteins, using the Cytoscape 3.6.0 editor and its plugins for analysis. The experimental design included the extraction of gene expression data related to five distinct types of cancers, namely, pancreatic ductal adenocarcinoma, hepatic cirrhosis, cervical cancer, glioblastoma, and anaplastic thyroid cancer from Gene Expression Omnibus (NCBI GEO) followed by pre-processing and normalization of the data using R &amp; Bioconductor. ExprEssence plugin was used for network condensation to identify differentially expressed genes across the gene expression samples. Gene Ontology (GO) analysis was performed to find out the over-represented GO terms in the network. In addition, pathway enrichment and functional module analysis of the protein-protein interaction (PPI) network were also conducted. Our results indicated NOTCH1, NOTCH3, FLCN, SOD1, SOD2, NF1, and TLR4 as upregulated proteins in different cancer types highlighting their role in cancer progression. The MCODE analysis identified gene clusters for each cancer type with *MYC*, *PCNA*, *PARP1*, *IDH1*, *FGF10*, *PTEN*, and *CCND1* as hub genes with high connectivity. *MYC* for cervical cancer, *IDH1* for hepatic cirrhosis, *MGMT* for glioblastoma and *CCND1* for anaplastic thyroid cancer were identified as genes with prognostic importance using survival analysis.

## Introduction

Rapamycin, also known as sirolimus, is a macrolide produced by *Streptomyces hygroscopicus* that targets mTOR (mechanistic target of rapamycin) and displays antifungal, anti-tumor, immunosuppressive and anti-aging characteristics [1, 2]. mTOR is an evolutionary, well-conserved serine/threonine protein kinase that belongs to the phosphoinositide3-kinase (PI3K)-related kinase (PIKK) family. It plays a pivotal role in regulating cell growth and metabolism in response to various environmental inputs such as growth factors and nutrients. Deregulation of mTOR-signaling network leads to various diseases such as cancer, diabetes and age-related disorders [1]. mTOR forms the catalytic subunit of two protein complexes, namely, mTOR complex 1 (mTORC1) and mTOR complex 2 (mTORC2) [3, 4].

**Fig. 1:**
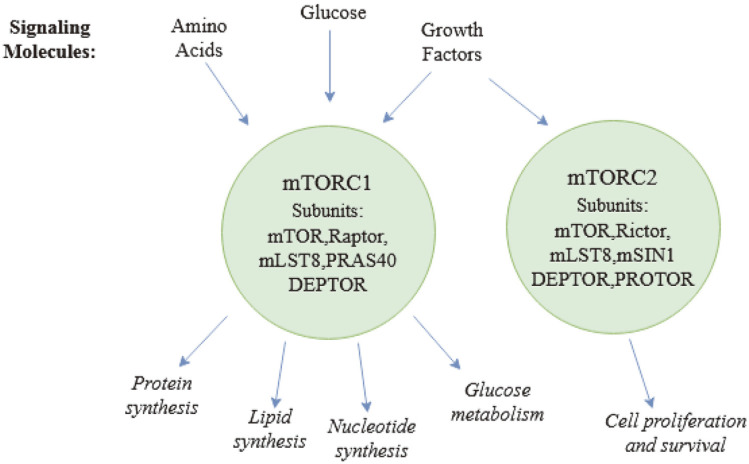
Signaling and function of complexes formed by mTOR Ser/Thr kinase: mTORC1 and mTORC2 and their respective subunits.

Rapamycin inhibits mTORC1, which comprises three core proteins: mTOR, Raptor, and GβL, and two inhibitory components, namely, PRAS40 and DEPTOR [5, 6]. mTORC1 promotes a balance between the anabolic and catabolic processes in cells, where on one hand it facilitates protein, lipid, and nucleotide synthesis and glucose metabolism as shown in Fig. 1; on the other it suppresses catabolism of proteins and autophagy [1]. mTORC1 promotes initiation of mRNA translation and hence protein synthesis by phosphorylation of S6K1 and eIF4E binding proteins [7, 8]. Synthesis of lipids is facilitated by mTORC1 through transcription factors such as sterol regulatory element-binding protein (SREBP) [9]. Purine and pyrimidine synthesis takes place when mTORC1 activates ATF4-dependent expression of MTHFD2 and carbamoyl phosphate synthetase (CAD) respectively [10]. Rheb is a crucial and effective activator of mTORC1 kinase activity in its GTP-bound state, which is negatively controlled by its GAP (GTPase activating protein), the tuberous sclerosis heterodimer TSC1/2 [11-13]. To regulate Rheb's nucleotide-loading state, most upstream inputs are routed through AKT and TSC1/2. Amino acids, on the other hand, signal to mTORC1 independently of the PI3K/Akt axis, promoting its translocation to the lysosomal surface, where it can be activated by Rheb [14]. Multiple complexes, including the v-ATPase, Ragulator, the Rag GTPase, and GATOR1/2, work together to modulate this process [15, 16].

mTORC2 is made up of the proteins mTOR, RICTOR, GβL, SIN1, PRR5/Protor-1, and DEPTOR [17]. By activating AKT, mTORC2 increases cellular survival, regulates cytoskeleton dynamics by activating PKCα, and controls ion transport and growth by phosphorylation of SGK1 [18-20]. Unlike mTORC1, mTORC2 does not react to rapamycin treatment because rapamycin FKBP12 complexes do not bind or directly inhibit mTORC2. However, prolonged treatment has been shown to obstruct mTORC2 signaling, probably because rapamycin bound to mTORC2 is unable to incorporate into new mTORC2 complexes [21].

mTOR signaling dysfunction is linked to a variety of diseases, including cancer, cardiovascular disease, neurodegenerative diseases, and diabetes. In human malignancies, many components of the PI3K signaling pathway, downstream of both mTORC1 and mTORC2, are mutated. Mutations in genes encoding proteins such as PTEN, TSC1/2, serine threonine kinase 11 (LKB1) and NF1 that lie upstream of the mTOR complexes generate cancer syndromes [22]. By limiting mTORC2-mediated AKT phosphorylation, mTOR kinase inhibitors can influence cell survival and proliferation [23]. The increased activity of RTK-PI3K-PDK1 in response to mTOR kinase inhibitors may revive AKT phosphorylation on Thr308 [24]. Activation of the mTORC2-AKT pathway lowers lipolysis and increases glucose absorption. In obesity, high levels of circulating nutrients and cytokines stimulate mTORC2 activity, which suppresses insulin signaling and leads to insulin resistance (IR) through a variety of pathways [25].

In our study, we examined five distinct microarray datasets, namely, GSE163031 (accessed in April 2021), GSE166163 (accessed in March 2021), GSE166466 (accessed in May 2021), GSE15824 (accessed in June 2021), and GSE53072 (accessed in April 2021), which belonged to pancreatic ductal adenocarcinoma (PDAC), hepatic cirrhosis, cervical cancer, glioblastoma (GBM) [26] and anaplastic thyroid cancer (ATC) [27] respectively. These datasets were downloaded from Gene Expression Omnibus (GEO). Both normal and tumor samples were included in these datasets. In addition, mTORC1 network was built using Natural Language Processing (NLP). This was followed by Gene Ontology (GO) and pathway enrichment studies along with a functional module analysis of protein-protein interaction (PPI) network which yielded significant genes and pathways linked to various malignancies. Cluster genes were identified and survival analyses of selected genes were also performed.

## Material and Methods

### Raw Biological Data

We retrieved 5 microarray datasets from NCBI (Gene Expression Omnibus) as shown in Table 1. These microarray datasets contained both normal and tumor samples across 5 different types of cancer and their platforms comprised of GPL19117, GPL23126, GPL570, and GPL6244. GEO include a functional genomics data repository that accepts MIAME-compliant data submissions. It contains data based on arrays and sequences, allowing researchers to download curated gene expression profiles for their studies [https://www.ncbi.nlm.nih.gov/geo].

### Data Preprocessing and DEGs Analysis

The preprocessing and normalization of raw biological data is the first step in DNA microarray data analysis. This procedure eliminates noise from biological data and ensures its consistency. The robust multi-array average (RMA) analysis tool in the affy package of R version 3.3.3 and Bioconductor was used to perform background correction, normalization, and summarization of probe data [28]. For preprocessing, CEL files and probe annotation files, also known as chip description files (CDF), were retrieved from Brain Array. Every dataset was preprocessed, resulting in a normalized text file containing gene expression values. The DEGs were screened out via the LIMMA package throughout the cut-off criteria of *p* < 0.01 and log2 (fold change) > 2.

### Construction of PPI Network Using NLP Methods

The information source for bioinformatics text mining was Medline/PubMed. The NCBI PubMed portal was used to retrieve Medline abstracts. We explored PubMed using the keywords (mTORC1 and disease) and the timeframe 2004-2019. A total of 6,097 studies were downloaded. The names of authors and their affiliations as well as the journal and year of publication were all included in the downloaded papers. As the first round of hits, text mining yielded 1401 genes. Considering that text mining systems are known to create a substantial number of false positives, we manually filtered these hits and used several filters to exclude gene names that matched common English words, acronyms, and technique keywords. Subsequently, using the Cytoscape 3.6.0 editor and associated plugins [29], we created an electronic network of mTORC1 with 206 nodes representing proteins and 470 edges denoting activations/interactions and/or inhibitions.

### Integration of Gene Expression Data from of 5 Different Types of Cancer into the PPI Network

Combining differential gene expression data with the network of regulation/interaction can assist researchers in figuring out which pathways are involved in switching states. To comprehend and emphasize the most significant start-ups and shut-downs in the process of cancer maintenance, we selected and preprocessed a number of gene expression datasets to condense the network to a collection of network linkages. Expression data were retrieved from GEO and preprocessed by the RMA technique. These data were then integrated into the network and condensed using the ExprEssence app (formerly called a ‘plugin’) of Cytoscape, which searches for differentially altered links in a given network using multiple sets of expression data [30]. In the condensed network, the top 10% of differentially altered links were considered for analysis. These are referred to as the most differentially changed linkages. Hypotheses about the initiation or termination of interactions, stimulations, and inhibitions are highlighted in the figures.

### Gene Ontology and Pathway Enrichment Analysis

**Fig. 2:**
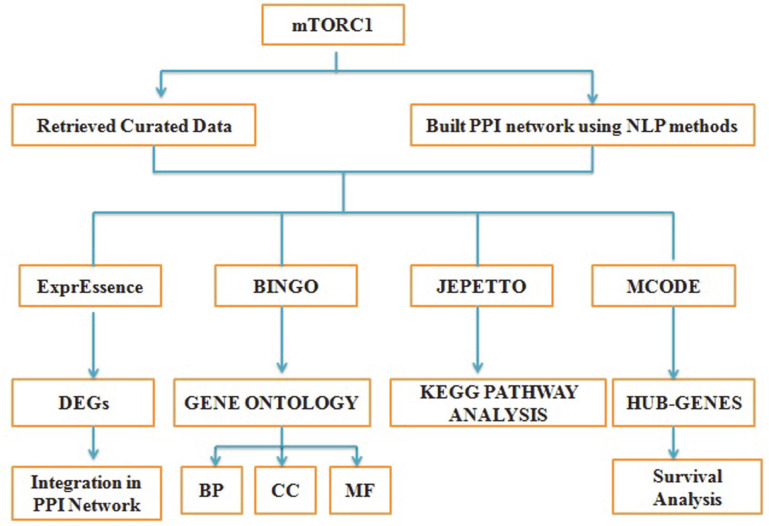
Flowchart depicting the overall methodology followed for the study.

The Biological Networks Gene Ontology tool, an expandable and adaptable Cytoscape plugin was used to conduct GO investigations as shown in Fig. 2. The GO terms were classified into three categories, including biological process (BP), cellular component (CC), and molecular function (MF). This plugin looks for GO terms that are over-represented in biological networks. The cut-off criterion for statistically over-represented GO terms was set at *p* < 0.05. The BiNGO can be used to discover which Gene Ontology (GO) concepts are considerably over-represented in a set of genes. BiNGO version 3.0.1 can be used interactively on subgraphs of biological networks visualized in Cytoscape or on a list of genes provided as text. BiNGO uses Cytoscapés extensible visualization environment to create an intuitive and adaptable visual representation of the results, mapping the prominent functional themes of the tested gene set on the GO hierarchy [31]. For pathway enrichment analysis, the Cytoscape plugin JEPETTO (Java Enrichment of Pathways Extended to Topology) version 1.3.1 was used. JEPETTO is a Cytoscape 3.x plugin that analyses human gene sets in an integrated manner. Using protein interaction networks and topological analysis, it identifies functional correlations between genes and known cellular pathways and functions [32]. In addition, *p* < 0.01 was used for identifying enriched pathways by KEGG pathway analysis.

In the reference database, there are overlapping pathways/processes, as seen in the ranking. They are sorted by the network-based association score (XD-score), and each overlap is given a statistical significance (q-value of the Fisher exact test adjusted for multiple testing using Benjamini and Hochberg procedure). XD-score assesses how close a pathway and the input gene set are in the molecular interaction network using a random walk in relation to the baseline model (average distance between the input set and all pathways). Positive XD-score values suggest a more intimate relationship than the average, while negative values imply a more distant relationship [33]. A mapping of the investigated gene set on the interaction network, as well as a regression plot of the XD-score/q-value relationship, are included in the extra tabs, which shows the significance threshold for XD-score. An extra tissue tab containing XD-scores for 60 human tissues appears if tissue-specific analysis is enabled. The molecular interaction network is restricted to the specific molecular interaction network for each score [34].

### Module Analysis Using MCODE

Molecular Complex Detection (MCODE) version 1.5.1 was used to screen modules of PPI networks with degree cut-off =2, node score cut-off =0.2, k-core cut-off =2, and max depth =100. A high k-core score implies a central location of the gene in the network. MCODE (Molecular Complex Detection) is a technique for detecting densely connected regions in huge protein-protein interaction networks that could be molecular complexes. To extract the dense regions according to provided parameters, the approach uses vertex weighting by local neighborhood density and outward traversal from a locally dense seed protein. The algorithm has the advantage of having a directed node, which permits fine-tuning of clusters of interest without considering the remainder of the network and investigation of cluster interconnectivity, which is important in protein networks [35].

### Survival Analysis

The Pathology Atlas (https://www.proteinatlas.org/humanproteome/pathology), which is part of the Human Protein Atlas, is based on a systems-based analysis of the transcriptome of 17 major cancer types using data from 8,000 patients and illustrates the impact of protein levels on cancer patient survival. It contains data on 291 patients with cervical cancer, 176 with pancreatic cancer, 365 with liver cancer, 153 with glioblastoma, and 501 with anaplastic thyroid cancer (ATC). The Pathology Atlas was utilized to estimate survival curves using Kaplan–Meier plots in order to find relevant genes associated with various malignancies. Clustered gene symbols were entered against their associated cancer type, and the findings were assessed using *p*-values [36].

## Results and Discussion

### Protein–Protein Network Construction

**Fig. 3:**
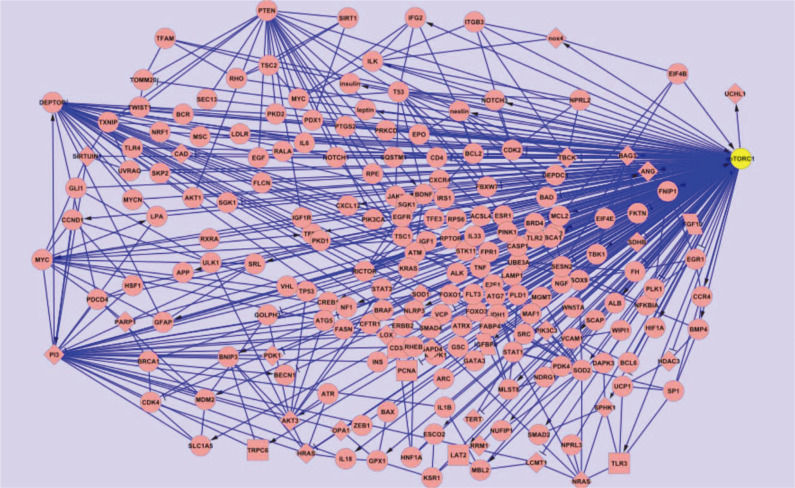
Electronic representation of mTORC1: The network consists of 206 nodes and 470 edges. The overall network is represented in the form of an electronic circuit depicting the nodes (genes/proteins) and the edges (activation/inhibition/interaction).

We manually added those nodes (genes/proteins) and edges (activations, interactions, inhibitions) to the mTORC1 network which have been documented in literature as mechanisms involved in induction and maintenance of the mTORC1 protein. We created an electronic network comprising 470 activations/interactions and/or inhibitions of mTORC1 and 206 nodes representing proteins as depicted in Fig. 3. The Cytoscape 3.6.0 editor and its analysis plugin were used to create these electronic circuit models. The layout consisted of nodes and edges, with nodes representing genes/proteins. For the edges, two types of mechanisms were considered: 1) activation shown by an arrow, and 2) inhibition denoted by a T-bar. Further nodes (genes/proteins) are annotated as enzymes (*e.g.*, carbonic anhydrase 1, phenylalanine hydroxylase, etc.), transcription factors (E2F transcription factor-1, heat shock transcription factor-1, etc.), or receptors (epidermal growth factor receptor, insulin-like growth factor-1 receptor, etc). mTORC1 activated 145 nodes (proteins/genes) explicitly and inhibited 24 nodes (proteins/genes).

### Visualization and Explorative Analysis of mTORC1 in Various Cancers

**Fig. 4:**
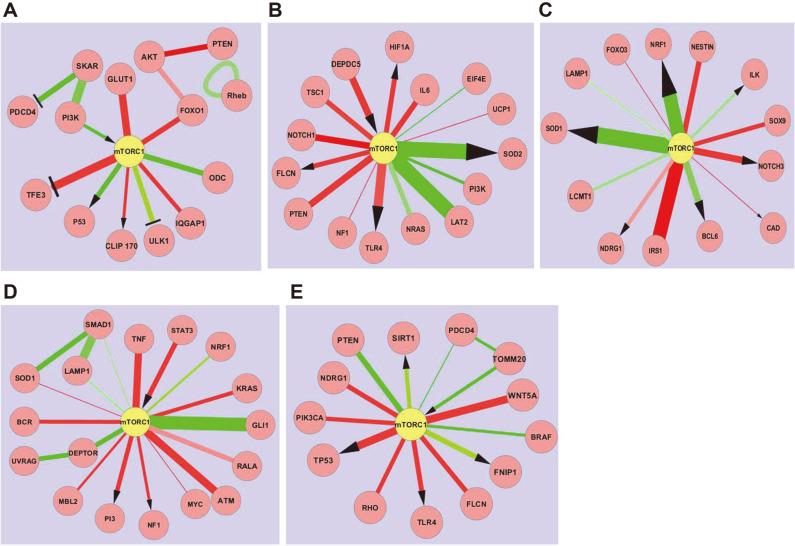
A network condensation through ExprEssence for A) Pancreatic Ductal Adenocarcinoma B) Hepatic Cirrhosis C) Cervical Cancer D) Glioblastoma E) Anaplastic Thyroid Cancer. Upregulated proteins/genes are denoted by red color edges and downregulated proteins/genes by green color edges. Arrows denote activation, T-bars denote inhibition, and straight lines denote interaction. The top 10% start-ups (red) and the top 10% shutdowns (green) are highlighted.

In this section, we noticed a lot of shut-down surrounding mTORC1, which emphasizes the importance of mTOR signaling in various types of cancer. Pancreatic ductal adenocarcinoma or PDAC is the most common form of pancreatic cancer with a poor prognosis and leads to mortality in most cases. Fig. 4A depicts that the proteins GLUT1, TFE3, FOXO1, IQGAP1, CLIP170, AKT were elevated in PDAC while ODC, ULK1, PI3K, PDCD4, SKAR were downregulated. Overexpression of GLUT1, which is the ubiquitous receptor for glucose, has been attributed to the increased consumption of glucose by cancer cells. GLUT1 stimulation of mTOR occurred through a Tuberous Sclerosis Complex (TSC) and AMP kinase-independent pathway and appeared to be involved in GLUT1-stimulated release of the mTOR regulator [37]. MiT/TFE transcription factors and mTORC1 are involved in a feedback loop where mTORC1 inhibits TFEB nuclear localization and function is an important transcription factor responsible for the regulation of different biological processes such as cell cycle, metabolism, and apoptosis [38]. IQGAP1 is a putative oncoprotein that binds to mTORC1, functions like a rheostat and regulates cytoskeleton dynamics. CLIP170 is a cytoplasmic linker protein that binds to microtubules and promotes the motility of pancreatic cancer cells. The present study shows a direct activation of CLIP170 by mTORC1. AKT is a well-known serine/threonine kinase in the PI3K/AKT signaling pathway and plays an essential role as a molecular target in cancer therapy [39]. ODC is a rate-limiting enzyme in the polyamine synthesis pathway that converts ornithine to putrescine. Though it has been reported to be hyperactive in most cancers, in the present study on PDAC, it was found to be downregulated [40]. This observation however needs to be further validated using wet lab experiments. ULK1 (UNC51-like kinase1) is responsible for initiation of the autophagy cascade. ULK1 is regulated by both mTORC1 and AMPK which inhibit and activate ULK1 respectively [41]. Fig. 4A also shows inhibition of ULK1 by mTORC1. PI3K/AKT/mTOR signaling is an important intracellular pathway that acts as a key regulator of cancer. In pancreatic cancer cells, mTORC1 caused lower levels of PI3K and p53. We observed that PI3K is underexpressed in PDAC [42], and a lower level of p53 was also found.

As seen in Fig. 4B, NOTCH1, TSC1, FLCN, TLR4, NF1, PTEN, DEPDC5, IL6 and HIF1A are upregulated and SOD2, LAT2, NRAS are downregulated proteins/genes in case of hepatic cirrhosis, which is the most common cause of hepatocellular carcinoma (HCC). Notch signaling plays an important role in liver tissue and the literature supports the carcinogenic role of Notch in the development of HCC. Overexpression of NOTCH1 has been observed in HCC cells and also in mouse models of HCC [43]. The same has been corroborated by our results as demonstrated in Fig. 4B. In the present study, we also observed that PTEN overexpression inhibited the AKT signaling pathway and activated the mTOR signaling pathway so that at the transcriptional and protein level it upregulates apoptosis-associated genes. Experimental studies show that the inactivation of PTEN gene may play a critical role in the development of HCC [44]. As shown in Fig. 4B, mTORC1 causes overexpression of FLCN, also called Folliculin, which functions as a GTPase-activating protein (GAP) of RagGTPases, thus playing an important role in lysosomal biogenesis [45]. mTORC1-driven Toll-like receptor 4 (TLR4) overexpression is also linked to tumor cell proliferation. In a hypoxic tumor microenvironment, hypoxia-inducible factors (HIF) are overexpressed which act on a variety of target genes modulating angiogenesis, metastasis, and cellular metabolism [46]. Mutations in DEPDC5, a component of the GATOR1 complex, cause persistent activation of mTORC1 leading to liver damage and development of HCC, as shown in Fig 4B. IL-6 (interleukin 6) is a cytokine that plays a main role in hematopoiesis, cell growth, and differentiation. As IL-6 is significantly overexpressed in HCC, it has been considered as a tumor marker for the disease [47]. SOD2 is a mitochondrial antioxidant and a key aging factor which is downregulated in HCC. NRAS (neuroblastoma RAS viral oncogene homolog) has been recently identified as a prognostic marker in HCC [48].

Cervical cancer is the second most common malignancy of women worldwide. Fig. 4C depicts SOX9, NDRG1, NESTIN, NOTCH3, IRS1, PDK1, and CAD as upregulated proteins and BCL6, ILK, SOD1, NRF1, LEPTIN as downregulated proteins in cervical cancer. Increased NESTIN expression suggests that NESTIN plays a significant role in carcinogenesis and tumor formation of cervical cells and helps in the regulation of CSC (cancer stem cell) function. NESTIN expression could also potentially be a useful marker in the early detection of cervical cancer. NOTCH can be a tumor suppressor but has an oncogenic role in many cancers. Upregulation of NOTCH in cervical cancer has been observed in previous studies [49]. SOX9 is SRY-box transcription factor-9 which is overexpressed in cervical cancer and acts as a potential therapeutic target. This overexpression of SOX9 activated p21 WAF1/CIP1 via a specific promoter region which basically blocks G1/S transition in the cell cycle [50]. PDK1 (pyruvate dehydrogenase kinase 1) is the main regulatory enzyme implicated in the metabolic reprogramming of tumors. Overexpression of PDK1 in cancer cells inhibits PDC-catalyzed TCA (tricarboxylic acid cycle) and resulting aerobic oxidation [61]. Overexpression of insulin-related substrate-1 activates the AKT and MAPK proteins, thus contributing to tumorigenesis. The downregulated protein NRF1 is an important transcription factor in the human genome and has been reported to regulate the expression of some key metabolic genes involved in cell growth. Fig. 4C depicts underexpression of ILK (integrin-linked kinase) which is a multifunctional serine/threonine kinase in the cytoplasm. Current studies indicate that cancer patients with increased ILK expression have low survival, poor prognosis and increased metastasis. However, not much is known about ILK proteins and the implication of their downregulation in cervical cancer [51]. The human papillomavirus which causes cervical cancer leads to severe oxidative stress in the human body. Superoxide dismutase (SOD) enzyme is a component of the ‘cellular antioxidant system’ and is crucial to maintaining the balance between formation and removal of reactive oxygen and reactive nitrogen species. Downregulation of SOD1 by mTORC1 in cervical cancer could lead to oxidative stress. Though BCL6 is reportedly overexpressed in stomach cancer, head and neck cancer, etc., Fig. 4C clearly shows downregulation of the same in cervical cancer. LCMT1 and LEPTIN are weakly downregulated in cervical cancer [52].

Glioblastoma (GBM) continues to be the most aggressive primary brain tumor in adults with high mortality within 1-2 years of diagnosis. As illustrated above in Fig. 4D, PI3, STAT3, TNF, KRAS, ATM, NF1, BCR are upregulated proteins for GBM while GLI1, DEPTOR, LAMP1, SMAD1 are downregulated. DEPTOR directly regulates UVRAG protein. According to studies, STAT3 (signal transducer and activator of transcription 3) is a potent regulator of gliomagenesis through its induction of angiogenesis, host immunosuppressant and tumor invasion. STAT3 acts as a target for inhibition in cancer therapy. Rahaman *et al*. observed constitutive activation of STAT3 in 90% of human GBM cell lines. STAT3 activity is connected with the upregulation of anti-apoptotic molecules such as Bcl-2, Mcl-1 and plays a role in cell survival. *NF-1* codes for a neurofibromin protein which is a RAS-GTPase that works as a tumor suppressor gene and is highly mutated in GBM. As shown in Fig. 4D, overexpression of NF1 was observed in the network. Recent studies have revealed that this protein also inhibits glioma invasion. K-RAS (Kirsten-rat sarcoma viral oncogene homolog) is an oncogene that drives tumorigenesis and its overexpression also leads to activation of mTORC1. Tumor necrosis factor (TNF) establishes a link between inflammation and cancer and its overexpression results in recruitment of NF-kB and STAT6 and invasion by glioma cells [53]. GLI1 functions as a transcriptional activator and is tightly regulated during embryo development and tissue differentiation. In differentiated tissues, GLI1 has low levels of expression which is also seen in Fig. 4D. However, in certain cancers, activation of GLI1 leads to various hallmarks of cancer such as proliferation, angiogenesis, and survival [54]. Lower levels of DEPTOR (Fig. 4D) would implicate regular functioning of mTORC1 pathway as DEPTOR is an inhibitor of mTORC1. UVRAG acts as a tumor suppressor and therefore its expression level is lower in glioblastoma. LAMP1 is lysosome-associated membrane protein that is slightly downregulated in GBM [55].

In anaplastic thyroid cancer (ATC), TP53, RHO, TLR4, WNT5A, and FLCN are overexpressed while PTEN, SIRT1, PDCD4, TOMM20, BRAF, and FNIP1 have a low level of expression. PDCD4 mediates TOMM20 protein as demonstrated in Fig. 4E. mTORC1 drives overexpression of TLR4, thereby leading to cell proliferation. TP53, guardian of the genome, is upregulated by mTORC1 (Fig. 4E) and a high rate of mutation reported in this gene has been related to the severe aggressiveness of ATC [56]. RHO protein, a GTPase that stimulates cell cycle progression and inhibits apoptosis was found to be overexpressed. It increases levels of cyclin D1 which has been reported to be linked with thyroid cancer aggressiveness [57]. The anaplastic subtype of thyroid cancer was significantly related to PTEN transcriptional silencing, implicated in the carcinogenesis of highly malignant or late-stage thyroid cancers. TOMM20 is involved in metabolic processes and Wnt5A behaves as a tumor suppressor, both of which have been observed to be downregulated in ATC in this study [58].

### Gene Ontology Enrichment Analysis

We performed gene enrichment analysis of the genes/proteins involved in the mTORC1 network to find the gene ontology (GO) terms over-represented into the biological process (BP), molecular function (MF), and cellular compartment (CC). The GO analysis was conducted for all 206 genes/proteins of the mTORC1 network using BiNGO. “GO slim generic” gene ontology reference annotation was used, which is a set of high-level GO terms and the results are depicted in Table 2. Positive regulation of biological and cellular processes and regulation of cell death and signaling pathways were identified as the most significant processes in the network. The majority of the proteins are localized in the cytosol, cytoplasm, intracellularly, and in various intracellular parts. In terms of function, receptor binding, kinase binding, receptor signaling protein activity and protein serine/threonine kinase activity they are over-represented. Similar functions have been attributed to the DEGs in the previous section using the ExprEssence app.

### KEGG Pathway Analysis

The JEPETTO plugin of Cytoscape was used for KEGG pathway enrichment analysis for the top module of nodes [http://apps.cytoscape.org/apps/jepetto]. KEGG enrichment analysis was conducted to understand the biological meaning of the DEGs. The top seven pathways that were significantly enriched included pathways involved in cancer, prostate cancer, pancreatic cancer, mTOR signaling, insulin signaling, neurotrophin signaling, and melanoma for the mTORC1 network as shown in Table 3. The data were imported into Cytoscape to calculate the topological characteristics of the network and determine the nodes. The KEGG database contains information on the progression of many types of cancer, as well as signaling pathway combinations. Signaling pathways are the chemical interactions and reactions that transport signals from the outside to the cell nucleus, where transcriptional regulation takes place. The MAPK, WNT, and TGF-beta signaling pathways, for example, have been extensively explored in the context of cell proliferation [59]. mTORC1 controls “insulin signaling” by regulating several downstream components such as growth factor receptor-bound protein 10 (GRB10), insulin receptor substrate (IRS-1), F-box/WD repeat-containing protein 8 (Fbw8), and insulin-like growth factor-1 receptor/insulin receptor (IGF-IR/IR). mTORC1 responds to glutamate and neurotrophin during neuronal development to enhance neuronal migration and dendritic arborization [60]. mTORC1 is also strongly linked with the pathogenesis of melanoma [61].

### Module Analysis Using MCODE

**Fig. 5:**
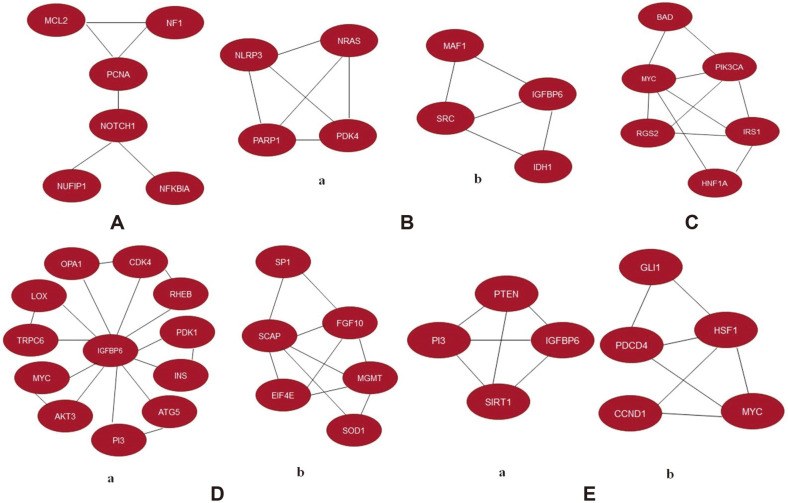
MCODE analysis for A) Pancreatic Ductal Adenocarcinoma B) Hepatic Cirrhosis C) Cervical Cancer D) Glioblastoma E) Anaplastic Thyroid Cancer. Cluster members are shown in red. MCODE parameters- degree cutoff- 2, node score cut-off-0.2, k-core cut-off-2 and max depth-100. One cluster was obtained for Pancreatic ductal adenocarcinoma and Cervical cancer where as two clusters each were obtained for Hepatic cirrhosis, Glioblastoma, and Anaplastic thyroid cancer.

MCODE hunts for clusters (regions with a multitude of connections) in a network. MCODE results for pancreatic ductal adenocarcinoma are depicted in Fig. 5A, which represents 6 nodes (NF1, MCL2, PCNA, NOTCH1, NUFIP1, and NFKBIA) and 7 connections. Proliferating cell nuclear antigen (PCNA) expression is high in pancreatic cancer cells, which is linked to a poor prognosis. PCNA acts as a mediator in the above cluster for interaction with other genes. NF1 is a RAS GTPase gene that aids in cell growth regulation whereas MCL2 regulates apoptosis. In pancreatic cancer, PANC-1 cells, silencing NFKBIA increased proliferation and migration [62].

MCODE resulted in 2 coherent clusters for hepatic cirrhosis with 4 nodes for each sub-network as illustrated in Fig. 5B. There are 10 interactions in total. PDK4, PARP1, NLRP3, and NRAS are the nodes for the first cluster as shown in Fig. 5B (a) whereas IDH1, SRC, MAF1, and IGFBP6 are the nodes for the second sub-network which is shown in Fig. 5B (b). By elevating hepatic insulin/Akt signaling and activating an AMPK/FOXO1/CD36 lipid regulatory axis, PDK4 inhibition promotes liver regeneration by reprogramming glucose and lipid metabolism [32]. The PARP pathway of reactive oxygen/nitrogen species is involved in the pathogenesis of liver inflammation, metabolism, and fibrosis. Autophagy and NLRP3 inflammasome have important roles in the fibrosis of the liver. The IL-1 family pathway is a critical modulator of liver damage and fibrogenesis since it constitutes the main downstream signaling of the NLRP3 inflammasome. NRAS has been reported to be a prognostic marker in HCC. Mutant IDH causes cancer by altering a variety of cellular processes such as histone demethylation and DNA modification. In liver cancer, SRC signaling is important for cellular proliferation, invasion, migration, angiogenesis, and treatment resistance [55]. PTEN's tumor suppressor and metabolic actions are driven by MAF1, a critical downstream target [44].

In case of cervical cancer, a cluster of 6 nodes, namely, BAD, MYC, IRS1, RGS2, PK3CA, and HNF1A with 10 links was obtained. Fig. 5C shows that MYC protein is interacting with each node in the cluster. The BAD and PK3CA genes appear to represent a connection between apoptotic pathways and growth factor receptor signaling. MYC is already reported as an oncogene while IRS1 is involved in insulin signaling, which is also observed to be upregulated in Fig. 4C [63].

Cluster prediction in glioblastoma resulted in 2 sub-networks with 12 and 6 nodes respectively. Each protein in Fig. 5D (a) sub-network interacts with insulin growth factor binding protein 6 (IGFBP6). Elevated expression levels of IGFBP6 have been directly correlated to the time of survival in GBM patients. This being a hub gene could have interesting implications in GBM biomarker identification and therapy. MYC and SOD1 have been observed as other proteins in the cluster, which have been reported as overexpressed as shown in Fig. 4D. FGF10 is seen to be the centralized protein for the second sub-network as shown in Fig. 5D (b). The level of this fibroblast growth factor has been reported to be high in several tumors. In glioblastoma, abnormal PAM signaling can enhance critical metastatic processes such as angiogenesis and immune cell regulation. Lysyl oxidase (LOX) secreted preferentially by PTEN-deficient cancer cells promotes recruitment of macrophages. Extensive reports have cited higher LOXL1 expression as being linked to more aggressive glioma development [55].

Module Analysis for anaplastic thyroid cancer also resulted in 2 sub-networks containing 4 and 5 nodes respectively. HSF1 is a centralized protein in the second sub-network as depicted in Fig. 5E (b). The first cluster contains SIRT1, PTEN, P13, IGFBP6 and CCND1, MYC, PDCD4, HSF1, GLI1 for the second cluster. All the proteins in the Fig. 5E (a) sub-network are interconnected to each other protein. Sirtuin family protein SIRT1, which reportedly plays an important role in maintaining homeostasis under genotoxic stress, is overexpressed in human thyroid tumors, and its levels are linked to those of the cytoplasmic MYC protein. PTEN deficit causes thyroid carcinogenesis, which is aided by SIRT1. Overexpression of IGFBP6 inhibited cancer cell proliferation, invasion, and metastatic activities while also increasing apoptosis [56]. Thyroid malignancies have abnormally high levels of the cell cycle regulator cyclin D1; however, CCND1 gene alterations are uncommon in these tumors.

### Survival Analysis

**Fig. 6:**
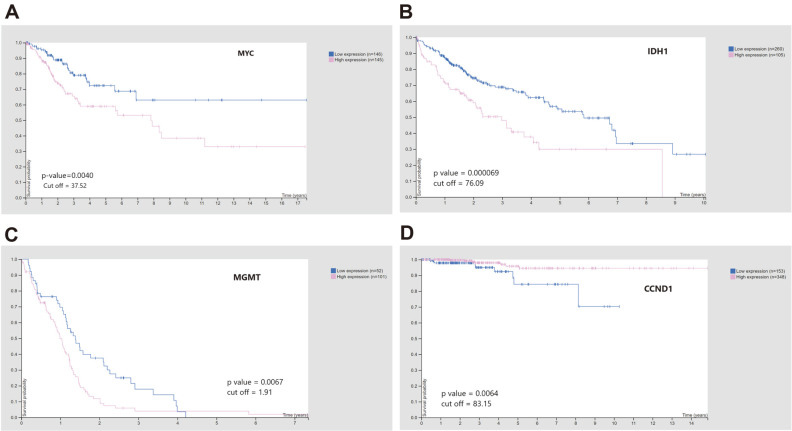
Kaplan-Meier plots for hub-genes: A) *MYC* B) *IDH1* C) *MGMT* D) *CCND1*. The blue color indicates low expression while pink color shows high expression.

Each gene associated in clusters for various malignancies was subjected to survival analysis and its prognostic value evaluated. Overall survival was substantially linked with *p*-values less than 0.01 [36]. In cervical cancer, MYC, a transcription factor which leads to upregulation of several genes involved in cell cycle progression, cellular transformation , and apoptosis was identified with a *p*-value of 0.004 (Fig. 6A). Kaplan-Meier plots of high significance were obtained in hepatic cirrhosis for *PARP1*, *PDK4*, and *NRAS*, *MAF1*, and *IDH1*. The plot for *IDH1* with the lowest *p*-value has been depicted in Fig. 6B. *LOX* and *MGMT* (Fig. 6C) genes have *p*-values of 0.0087 and 0.0067, respectively, for glioblastoma. Expression and methylation status of DNA repair enzyme *MGMT* enables its use as a clinical biomarker in GBM [57]. For anaplastic thyroid cancer, *PTEN* and *CCND1* (Fig. 6D) show low *p*-values less than 0.01. However, pancreatic cancer module genes do not yield significant *p*-values. The blue-colored line in the Kaplan-Meier plots indicates low expression of the gene whereas pink color shows high expression.

In summary, considering the pivotal role played by mTORC1 and the associated metabolic pathways in the progression of various types of cancers, an elaborate network was constructed with 206 nodes and 470 edges using Cytoscape 3.6.0. This network was built using Natural language processing of existing literature on mTORC1 in various diseases. Microarray datasets containing normal and diseased samples of five different types of cancers, namely, pancreatic ductal adenocarcinoma (PDAC), hepatic cirrhosis, cervical cancer, glioblastoma (GBM), and anaplastic thyroid cancer were processed, DEGs were identified and integrated into the original network. Various plugins/apps in Cytoscape, such as, ExprEssence, MCODE etc., were used to assess the applicability of the network in different cancer types. Given two experimental datasets, ExprEssence condenses networks to only include the links between genes/proteins that have a significant amount of change in expression values. The results have clearly shown the directionality of interaction (activation or inhibition) between two proteins, mostly involving mTORC1 at one end and transcription factors, enzymes, and receptors on the other. Superoxide dismutase (SOD) family of proteins and neurofibromin (*NF1*) were found to be overexpressed in hepatic cirrhosis and GBM while NOTCH family was found overexpressed in HCC and cervical cancer types. Elevated levels of folliculin or FLCN and TLR-4 were observed in HCC and ATC. Some proteins are activated by mTORC1 directly; others have been previously reported as biomarkers or targets in various cancers. Functional enrichment and pathway analysis yielded a multitude of genes/proteins and pathways which have been reported to play a major role in mTORC1 signaling cascade in the progression of different cancer types. Cluster identification from the network and survival analysis led to the identification of genes/proteins with prognostic importance, namely, *MYC* in cervical cancer, *IDH1* in hepatic cirrhosis, *MGMT* in glioblastoma and *CCND1* in anaplastic thyroid cancer. In summary, our studies have used an integrated bioinformatics approach to build and characterize an mTORC1 network using NLP and also identified genes/proteins which have prognostic importance in five different cancer types.

mTORC2 has also been reported to play a crucial role in various diseases, though the available experimental evidence for the same is less than that for mTORC1. The major thrust of all studies on mTOR is to design better therapeutic molecules. Although rapamycin, its analogs and other inhibitors of mTOR are known, their utility is restricted owing to toxicity and lack of specificity. It is imperative to identify other targets in the mTOR network and design drugs for the same. Therefore, further studies on the role of both mTORC1 and mTORC2 in the progression of cancer are warranted.

## Figures and Tables

**Table 1:** NCBI GEO datasets used in this study.

GEO ID	Cancer type	Sample	Normal	Tumor	Platform
GSE163031	Pancreatic ductal adenocarcinoma	15	3	12	GPL19117
GSE166163	Hepatic cirrhosis carcinoma	6	3	3	GPL23126
GSE166466	Cervical cancer	20	7	13	GPL23126
GSE15824	Glioblastoma	45	5	40	GPL570
GSE53072	Anaplastic thyroid cancer	9	4	5	GPL6244

**Table 2:** Top twelve significantly enriched GO terms for mTORC1 network including biological processes, cellular components and molecular function using BiNGO.

Gene Ontology Term	Description	Corrected *p*-value	Gene names	Gene count
BP(Biological Process)	Positive regulation of biological process	9.3966E-38	APP|EPO|IRS1|UBE3A|TNF|CCND1|MYC|AKT1|SOX9|PDK1|DAPK3|RPS6|TSC1|HNF1A|TP53|SQSTM1|NOTCH3|VCP|NOTCH1|GATA6|FOXO3|PLD1|HIF1A|FOXO1|SCAP|NLRP3|MARK4|ATG7|SMAD2|EGR1|SMAD4|VCAM1|BDNF|INSR|PLK1|BRAF|IGF1|ESR1|BMP4|NFKBIA|IL6|CXCL12|BCL6|CDK4|SP1|CDK2|BCL2|MDM2|NF1|ATM|ATR|FGF10|FLT3|ITGB3|PTEN|ILK|BRCA1|GLI1|RPTOR|OPA1|CASP1|MLST8|JAK2|CCR4|HRAS|MBL2|GPX1|SPHK1|TFE3|TFEB|NGF|SIRT1|LAT2|CREB1|IL1B|ANG|TLR4|TLR3|TLR2|BECN1|FH|RALA|NUFIP1|SRC|PTGS2|EGFR|INS|NRAS|RXRA|ERBB1|HSF1|ERBB2|E2F1|MAPK1|RICTOR|VHL|EIF4E|STAT1|BAD|EGF|BNIP3|STAT3|SOD2|SOD1|CD4|GOLPH3|PINK1|FABP4|BAX|KRAS	110
	Positive regulation of cellular process	1.2987E-37	APP|EPO|IRS1|UBE3A|TNF|CCND1|MYC|AKT1|SOX9|PDK1|DAPK3|RPS6|TSC1|HNF1A|TP53|SQSTM1|NOTCH3|VCP|NOTCH1|GATA6|FOXO3|PLD1|HIF1A|FOXO1|SCAP|NLRP3|MARK4|ATG7|SMAD2|EGR1|SMAD4|VCAM1|BDNF|INSR|PLK1|BRAF|IGF1|ESR1|BMP4|NFKBIA|IL6|CXCL12|BCL6|CDK4|SP1|CDK2|BCL2|MDM2|NF1|ATM|ATR|FGF10|FLT3|ITGB3|PTEN|ILK|BRCA1|GLI1|RPTOR|OPA1|CASP1|MLST8|JAK2|HRAS|MBL2|GPX1|SPHK1|TFE3|TFEB|NGF|SIRT1|CREB1|IL1B|ANG|TLR4|TLR3|TLR2|BECN1|RALA|NUFIP1|PTGS2|EGFR|INS|NRAS|RXRA|ERBB1|ERBB2|E2F1|MAPK1|RICTOR|VHL|EIF4E|STAT1|BAD|EGF|BNIP3|STAT3|SOD2|SOD1|CD4|GOLPH3|PINK1|FABP4|BAX|KRAS	105
	Regulation of cell death	9.2454E-33	APP|EPO|PTEN|ILK|BRCA1|TNF|DEPTOR|CASP1|AKT1|SOX9|JAK2|HRAS|GPX1|SPHK1|DAPK3|RPS6|NGF|SIRT1|ARC|CREB1|PIK3CA|IL1B|TP53|TLR4|SQSTM1|TLR2|BECN1|VCP|NOTCH1|HDAC3|GATA6|PTGS2|FOXO3|EGFR|FOXO1|INS|NRAS|RXRA|TERT|BAG3|ERBB1|ERBB2|NLRP3|MAPK1|VHL|MARK4|ATG5|SMAD4|MGMT|STAT1|BDNF|BAD|BNIP3|BRAF|IGF1|SOD2|ESR1|SOD1|BMP4|NFKBIA|IL6|PINK1|BCL6|ALB|BCL2|NF1|BAX|ATM|KRAS	69
	Regulation of signaling pathway	1.3853E-32	APP|EPO|IRS1|ITGB3|PTEN|ILK|UBE3A|GLI1|TNF|RPTOR|UCHL1|STK11|DEPTOR|CCND1|OPA1|CASP1|MLST8|AKT1|JAK2|HRAS|GPX1|SPHK1|PRKCD|TSC2|TSC1|HNF1A|SIRT1|BCR|IL1B|ULK1|TP53|TLR4|SQSTM1|TLR3|TLR2|NOTCH1|HDAC3|FPR1|CXCR4|HIF1A|EGFR|FOXO1|INS|NRAS|GSC|ERBB1|ERBB2|RICTOR|SMAD2|SMAD4|EGF|INSR|BRAF|IGF1|ESR1|SOD1|BMP4|NFKBIA|TBCK|IL6|CD4|GOLPH3|PINK1|BCL6|SP1|MDM2|NF1|PDCD4|ATM|KRAS|ATR|EZH2|FGF10	73
CC(Cellular Component)	Cytosol	6.5687E-14	IRS1|PTEN|ILK|UBE3A|GLI1|RPTOR|CCND1|MLST8|AKT1|JAK2|HRAS|PDK1|GPX1|SPHK1|PRKCD|RPS6|CAD|TSC2|TSC1|ARC|PIK3CA|ULK1|PIK3C3|TP53|SQSTM1|VCP|SRC|FOXO3|FOXO1|BAG3|MAPK1|RICTOR|VHL|EIF4E|EIF4B|SMAD2|SMAD4|BAD|INSR|PLK1|SOD1|NFKBIA|GOLPH3|PINK1|FABP4|CDK4|RHEB|FASN|CDK2|BCL2|MDM2|PDCD4|BAX|EIF4G1	54
	Cytoplasm	6.5687E-14	APP|IRS1|UBE3A|TOMM20|TNF|STK11|CCND1|LAMP1|AKT3|SESN2|PDK4|AKT1|PDK1|FBXW7|DAPK3|PRKCD|RPS6|TSC2|ACSL4|TSC1|HNF1A|SDHB|ARC|TFAM|ULK1|ERG|TP53|SQSTM1|VCP|NOTCH1|FPR1|SLC1A5|FOXO3|PLD1|HIF1A|NDRG1|FOXO1|TERT|BAG3|SCAP|NLRP3|IGFBP6|MARK4|MSC|ATG7|ATG5|SMAD2|EGR1|UVRAG|SMAD4|BDNF|INSR|PLK1|IDH2|BRAF|IGF1|ESR1|GFAP|BMP4|NFKBIA|CDK4|RHEB|SP1|FASN|ALB|CDK2|BCL2|MDM2|NF1|NOX4|ATM|ATR|EIF4G1|ITGB3|PTEN|ILK|BRCA1|GLI1|PKD2|RPTOR|FLCN|UCHL1|OPA1|CASP1|MLST8|JAK2|MDP1|CCR4|CD34|HRAS|GPX1|KSR1|SPHK1|CAD|TFEB|NGF|SIRT1|SIRT3|LAT2|CREB1|PIK3CA|IL1B|PIK3C3|SGK1|TLR4|TLR3|TLR2|BECN1|FH|NUFIP1|HDAC3|SRC|UCP1|CXCR4|PTGS2|SRL|EGFR|NRAS|ERBB1|HSF1|ERBB2|E2F1|MAPK1|RICTOR|VHL|FNIP1|LDLR|EIF4E|EIF4B|BRD4|FKTN|RRM1|STAT1|BAD|EGF|BNIP3|STAT3|SOD2|SOD1|CD4|GOLPH3|PINK1|FABP4|PDCD4|BAX|KRAS	146
CC (Cellular Component)	Intracellular	3.8176E-13	APP|IRS1|UBE3A|TOMM20|TNF|TGM1|STK11|DEPTOR|CCND1|LAMP1|MYC|AKT3|SESN2|PDK4|AKT1|OIP5|SOX9|SKP2|MAF1|PDK1|FBXW7|DAPK3|PRKCD|RPS6|TSC2|ACSL4|TSC1|ESCO_2_|HNF1A|SDHB|BCR|ARC|MYCN|LOX|TFAM|ULK1|ERG|TP53|SQSTM1|NOTCH3|VCP|NOTCH1|FPR1|GATA6|TWIST1|GATA3|NRF1|SLC1A5|FOXO3|PLD1|HIF1A|NDRG1|FOXO1|TERT|BAG3|SCAP|NLRP3|IGFBP6|MARK4|MSC|ATG7|ATG5|SMAD2|EGR1|UVRAG|SMAD4|BDNF|INSR|PLK1|IDH2|BRAF|IGF1|ESR1|GFAP|BMP4|NFKBIA|TBCK|BCL6|CDK4|RHEB|SP1|FASN|ALB|CDK2|BCL2|MDM2|NF1|UBA3|NOX4|ATM|ATR|EZH2|EIF4G1|FGF10|ITGB3|PTEN|ILK|BRCA1|GLI1|PKD2|RPTOR|FLCN|UCHL1|OPA1|CASP1|MLST8|JAK2|MDP1|CCR4|CD34|HRAS|SEC13|GPX1|PARP1|KSR1|SPHK1|TFE3|ATRX|CAD|TFEB|NGF|SIRT1|SIRT3|LAT2|CREB1|PIK3CA|IL1B|ANG|PIK3C3|SGK1|TLR4|TLR3|TLR2|BECN1|FH|RALA|PCNA|NUFIP1|HDAC3|SRC|UCP1|CXCR4|NEDD8|PTGS2|SRL|EGFR|NRAS|RXRA|GSC|ERBB1|HSF1|ERBB2|RHO|E2F1|MAPK1|RICTOR|VHL|FNIP1|LDLR|EIF4E|EIF4B|BRD4|FKTN|RRM1|MGMT|STAT1|BAD|EGF|BNIP3|STAT3|SOD2|SOD1|CD4|GOLPH3|PINK1|FABP4|PDCD4|BAX|KRAS	179
	Intracellular part	1.7969E-12	APP|IRS1|UBE3A|TOMM20|TNF|TGM1|STK11|CCND1|LAMP1|MYC|AKT3|SESN2|PDK4|AKT1|OIP5|SOX9|SKP2|MAF1|PDK1|FBXW7|DAPK3|PRKCD|RPS6|TSC2|ACSL4|TSC1|ESCO_2_|HNF1A|SDHB|ARC|MYCN|LOX|TFAM|ULK1|ERG|TP53|SQSTM1|NOTCH3|VCP|NOTCH1|FPR1|GATA6|TWIST1|GATA3|NRF1|SLC1A5|FOXO3|PLD1|HIF1A|NDRG1|FOXO1|TERT|BAG3|SCAP|NLRP3|IGFBP6|MARK4|MSC|ATG7|ATG5|SMAD2|EGR1|UVRAG|SMAD4|BDNF|INSR|PLK1|IDH2|BRAF|IGF1|ESR1|GFAP|BMP4|NFKBIA|BCL6|CDK4|RHEB|SP1|FASN|ALB|CDK2|BCL2|MDM2|NF1|UBA3|NOX4|ATM|ATR|EZH2|EIF4G1|FGF10|ITGB3|PTEN|ILK|BRCA1|GLI1|PKD2|RPTOR|FLCN|UCHL1|OPA1|CASP1|MLST8|JAK2|MDP1|CCR4|CD34|HRAS|SEC13|GPX1|PARP1|KSR1|SPHK1|TFE3|ATRX|CAD|TFEB|NGF|SIRT1|SIRT3|LAT2|CREB1|PIK3CA|IL1B|ANG|PIK3C3|SGK1|TLR4|TLR3|TLR2|BECN1|FH|PCNA|NUFIP1|HDAC3|SRC|UCP1|CXCR4|NEDD8|PTGS2|SRL|EGFR|NRAS|RXRA|GSC|ERBB1|HSF1|ERBB2|RHO|E2F1|MAPK1|RICTOR|VHL|FNIP1|LDLR|EIF4E|EIF4B|BRD4|FKTN|RRM1|MGMT|STAT1|BAD|EGF|BNIP3|STAT3|SOD2|SOD1|CD4|GOLPH3|PINK1|FABP4|PDCD4|BAX|KRAS	175
MF(Molecular Function)	Receptor binding	5.2072E-8	PP|RALA|EPO|IRS1|SRC|ITGB3|PTEN|ILK|BRCA1|PKD2|TNF|INS|UCHL1|RXRA|OPA1|ERBB2|JAK2|PDK1|MBL2|SMAD2|IL33|VCAM1|BDNF|EGF|INSR|IGF1|NGF|BMP4|IL6|CD4|CXCL12|IL1B|UBA3|ANG|SQSTM1|FGF1	36
	Kinase Binding	5.2072E-8	IRS1|FLT3|BAD|INSR|PLK1|STAT3|BRAF|FOXO3|PLD1|CD4|CCND1|MAPK1|JAK2|TP53|TLR4|SQSTM1|PDK1	17
	Receptor signaling protein activity	6.5239E-8	SMAD2|SMAD4|STAT1|IRS1|EGF|INSR|PLK1|STAT3|BRAF|NGF|EGFR|ERBB1|ERBB2|MAPK1|JAK2	15
	Protein serine/threonine kinase activity	1.1786E-7	KSR1|DAPK3|PRKCD|PLK1|ILK|BRAF|PKD2|EGFR|BCR|STK11|PINK1|CDK4|ERBB1|AKT3|CDK2|AKT1|MAPK1|ULK1|ATM|SGK1|MARK4|PDK1|ATR	23

**Table 3:** Top seven enriched KEGG pathway analysis using JEPETTO plugin for mTORC1.

Pathway or Process	XD-score	q-value	Overlap /size
Pathways in cancer	0.31512	0.0000	44/304
Prostate cancer	0.90243	0.0000	22/84
Pancreatic cancer	0.95851	0.0000	20/70
mTOR signaling pathway	1.34621	0.00000	18/49
Insulin signaling pathway	0.29953	0.00000	18/123
Neurotrophin signaling pathway	0.43187	0.00000	18/121
Melanoma	0.92974	0.00000	17/123
